# Hybrid Mathieu Urethroplasty vs. Tubularized Incised Plate Urethroplasty for the Management of Distal Penile Hypospadias With a Small Glans

**DOI:** 10.3389/fped.2022.876791

**Published:** 2022-04-05

**Authors:** Mazen Omar Kurdi, Nagi Ibrahim Eldessouki, Mohammad Gharieb Khirallah

**Affiliations:** ^1^Pediatric Surgery Unit, King Abdulaziz University Hospital, Jeddah, Saudi Arabia; ^2^Pediatric Surgery Unit, Tanta University Hospital, Tanta University, Tanta, Egypt

**Keywords:** distal penile hypospadias, hybrid Mathieu urethroplasty, tubularized incised plate urethroplasty, small glans, meatal stenosis

## Abstract

**Introduction:**

Distal hypospadias is a common anomaly. Different surgical techniques have evolved through the years to manage this anomaly. Several factors may affect the prognosis. One of them is glans size. We compared the hybrid Mathieu urethroplasty (HMU) and the tubularized incised plate urethroplasty (TIPU) for the management of distal hypospadias with a small glans.

**Methods:**

Sixty-eight patients with distal hypospadias were included and categorized into two groups. Group A (*n* = 33) and group B (*n* = 35) patients were treated by HMU and TIPU, respectively. All patients had a small glans. In group A, the patients underwent Mathieu urethroplasty plus a deep incision of the urethral plate. In group B, the patients underwent TIPU. Urethral stents were used in all cases. Hypospadias objective score evaluation (HOSE) was used to assess the results.

**Results:**

Urethrocutaneous fistulae developed in two cases in group A and six cases in group B. Meatal stenosis was significantly lower (one case in group A vs. eight cases in group B). Glanular dehiscence occurred in two cases in group A and five cases in group B. The small glans strongly correlated with the development of both urethrocutaneous fistulae and meatal stenosis where the odd ratios were 3.500 (1.383–7.879) and 9.481 (1.114–12.669), respectively.

**Conclusion:**

Both techniques showed efficacy during management of patients with a small glans. HMU had better outcomes, shorter duration of stent and lesser incidence of complications than TIPU. Small glans was significantly related to urethrocutaneous fistulae and meatal stenosis in group B.

## Introduction

The repair of a distal hypospadias remains a hot topic of debate among pediatric surgeons. No single technique had been sufficient to manage the problem. Several techniques with several principles arising from the continually evolving understanding of the pathogenesis and surgical anatomy of the anomaly have been used to reconstruct the deficient part of the urethra (1).

Two main techniques had gained popularity during the past years, the perimeatal-based flap (Mathieu) and tubularized incised plate urethroplasty (TIPU). Both have a considerable success rate and a reasonable complication rate (2).

Hybrid Mathieu urethroplasty (HMU), a modification of the classic Mathieu urethroplasty, was used to increase the scope of the procedure by including patients with distal hypospadias and a small glans (3).

Several measures were applied to overcome the small size of the glans during TIPU. One of them is the usage of testosterone, either local or systemic, to increase the size of the penis. Another option is the grafting of the urethral plate (4).

We aimed at comparing the results of HMU and TIPU in the management of patients with distal hypospadias and a small glans.

## Patients and Methods

### Study Design

This is a randomized control trial that was conducted in a tertiary level hospital from January of 2012 to February of 2021. It was approved by an Institutional Review Board with the approval code 34403/1/21. The trial was registered in clinicaltrials.gov registry under identifier NCT04946058 clinicaltrials.gov/ct2/show/NCT04946058

All cases with distal hypospadias and a small glans (the width of the glans is <14 mm) were included. These measures were taken in the same way as Bush et al. did (4).

We excluded cases of proximal hypospadias, redo cases, cases with a normal-sized glans, and cases that used preoperative testosterone either in the form of a cream or an intramuscular injection.

Sixty-eight cases met our inclusion criteria. They were randomly categorized into groups A and B. The randomization was achieved by the closed envelop method.

### Operative Technique

**Figure 1:**
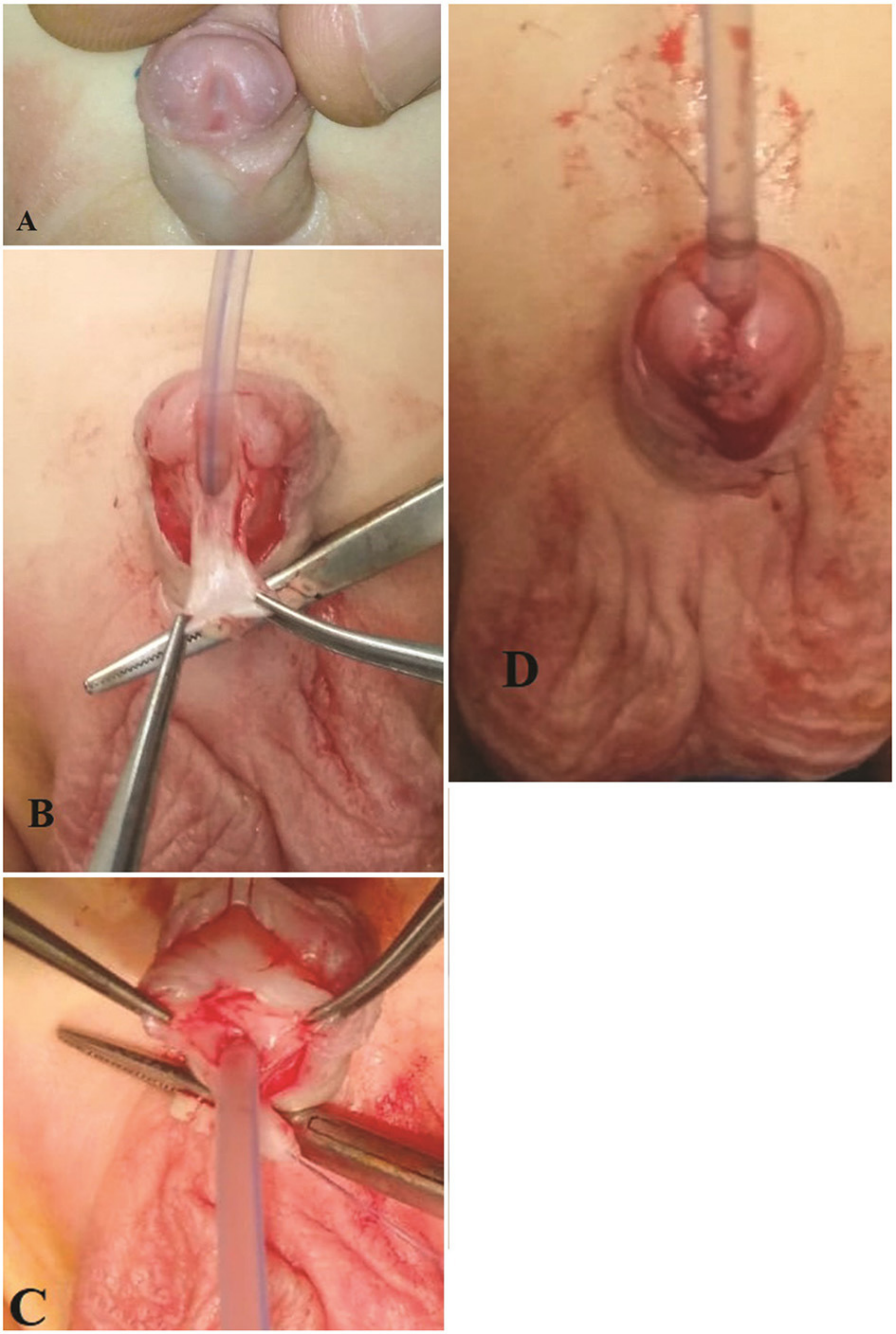
A case of HMU. **(A)** The Width of the glans is 11 mm. **(B)** Tailoring the perimeatal. lap, the black line showed the site and extension of the incision of the plate. **(C)** Incision of the urethral plate. **(D)** Complete reconstruction of the neourethra.

All procedures were performed by the three authors using magnifying devices (power ranged from 2.5 to 4). In group A (*n* = 33), the patients were operated on using the HMU. After the induction of anesthesia, the width of the glans was measured across the widest points of the glans before a stay suture was placed in the glans. This measure served as an index of the size of the glans. A stay suture was then taken through the glans. The perimeatal flap was created, and we performed adequate and extensive mobilization of the glanular wings. This was followed by a deep incision of the urethral plate extending from the meatus to just before the end of the plate. The neourethra was fashioned around the stent using 6/0 PDS sutures with a round needle tip in subcuticular manner. The glans and the skin of the shaft was closed using Vicry^©^ 5/0 with a round needle. The stent was secured in place. Topical broad-spectrum antibiotics and dressings were applied (Figure 1).

**Figure 2:**
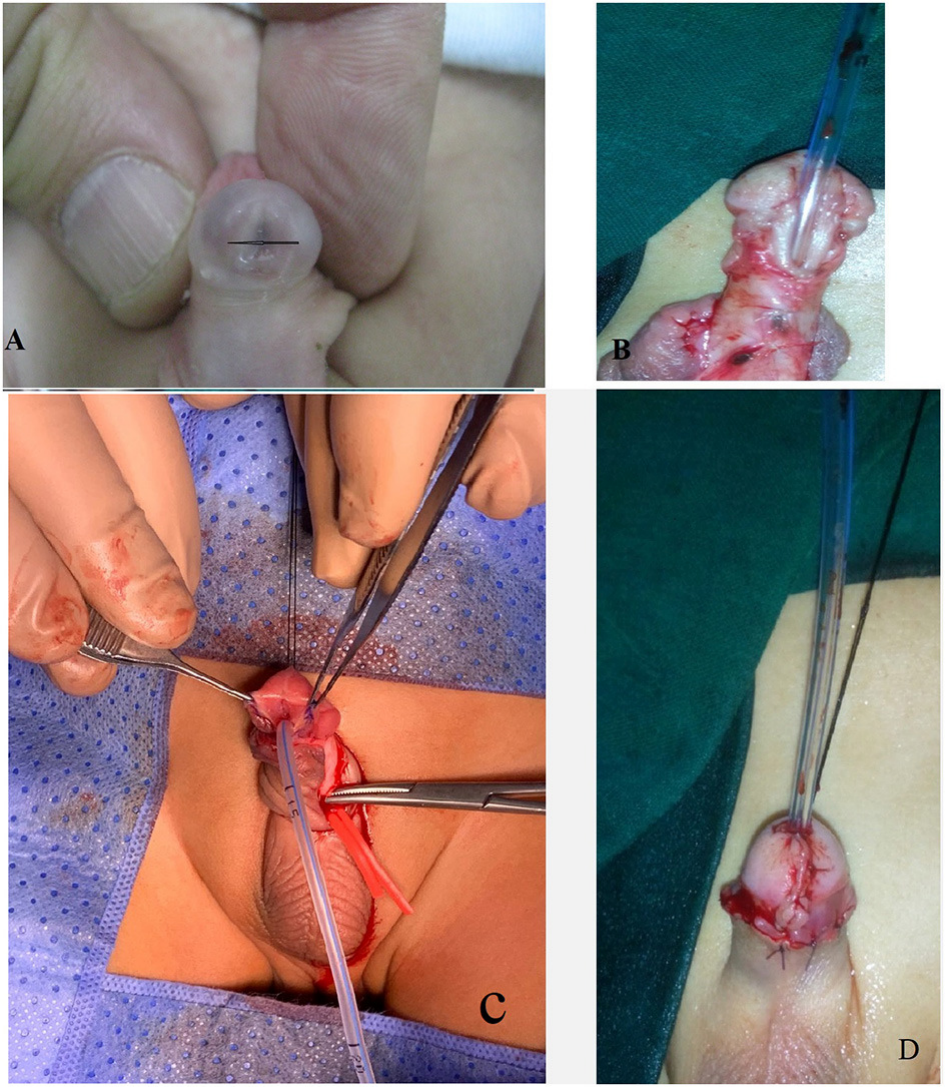
A case of TIPU. **(A)** The width of the glans is 10 mm. **(B)** Degloved penis and tailoring of the *U*-shape incision. **(C)** Incision of the plate. **(D)** Completing the procedure.

In group B (*n* = 35), the patients were operated on using the standard TIP urethroplasty. The same initial steps as in the group A were performed. This was followed by making incisions for the standard TIP urethroplasty. The neourethra was created using 6/0 PDS sutures with a round needle tip. An investing layer from the inner prepuce was taken to cover the suture line. The closure of the glans and the circumcision was performed. The stent was secured in place. Topical broad-spectrum antibiotics and dressings were applied (Figure 2).

Recorded data included the median age in years, median width of the glans in mm as an index of its size, mean operative time in minutes, mean duration of stents in days, the occurrence of postoperative urethrocutaneous fistula (UCF), meatal stenosis, meatal regression, glanular dehiscence and the success rates. All cases were followed up for at least 6 months. The results of the intervention in both groups were assessed by the hypospadias objective score evaluation (HOSE) (5).

#### Statistical Analysis

Statistical analysis was conducted via the SPSS ™ Statistics V21 (IBM SPSS, NY, USA). Numerical data were compared using an independent sample *t*-test, while categorical data were compared via a chi-square test, *p*-value < 0.05 was considered statistically significant. Quantitative variables were expressed by the median. Multivariate logistic regression was used to assess the presence or absence of UCF, glans dehiscence, meatal stenosis, and meatal regression when correlated with the size of the glans.

## Results

Our study included 68 patients with distal penile hypospadias. The patients were randomly categorized into two groups, A (*n* = 33) and B (*n* = 35). The median age in group A was 1.5 years (ranged from 1 to 2.5 years), while it was 1.25 years (ranged from 0.75 to 3 years) in group B. The median width of the glans was 11 mm (ranged from 9 to 14 mm) in group A and 12 mm (ranged from 10 to 14 mm) in group B. The position of the meatus was coronal in 23 cases (69.6%), subcoronal in 9 cases (27.3%), and anterior penile in a case (3%) in group A. In group B, the position of the meatus was coronal in 25 cases (71.4%), subcoronal in eight cases (22.8%), and anterior penile in two cases (5.7%). Five cases (15%) in group A and 4 cases (11.4%) in group B were circumcised (Table 1).

**Table 1:** Demographic, preoperative, and postoperative data in both groups.

	**Group A (*n* = 33)**	**Group B (*n* = 35)**	***p*-value**
Age at operation (median in years)	1.5 (1–2.5)	1.25 (0.75–3)	0.361
The width of the glans (median in mm)	11 (9–14)	12 (10–14)	0.273
**Position of the meatus (No. of cases)**			
- Coronal	−23 (69.6%)	−25 (71.4%)	0.517
- Subcronal	−9 (27.3%)	−8 (22.8%)	
- Anterior penile	−1 (3%)	−2 (5.7%)	
**Fore skin**			
- Present	−28 (84.8%)	−31 (88.5%)	0.153
- Circumcised	−5 (15.2%)	−4 (11.5%)	
Operative time (mean in minutes)	67.5 ± 15.2	79.2 ± 14.3	0.05^*^
Reconstruction of the fore skin (no. of cases)	No	31	–
Duration of urethral stent (median in days)	3 (2–5)	6 (4–8)	0.05^*^

* *Significant*.

The mean operative time was 67.5 min (±15.2 min) in group A vs. 79.2 min (±14.3 min) in group B. Reconstruction of the foreskin was performed in 20 cases in group A. However, reconstruction of the fore skin was performed for all uncircumcised patients in group B (Table 1).

The urethral stent was applied for a median of 3 days (ranged from 2 to 5 days) in group A, while it was in place for a median of 6 days (ranged from 4 to 8 days) in group B.

UCF developed in 2 (6%) cases in group A and 6 (17.1%) cases in group B, which was statistically significant. Glanular dehiscence was present in 2 (6%) and 5 (14.2%) cases in group A and group B respectively. The incidence of meatal stenosis was significantly lower in group A which had one (3%) case, while group B had 8 (22.9%) cases. These cases were subjected to dilatation and completely improved after frequent dilatation for 2 months. Meatal regression occurred in 2 (6%) cases in group A while it occurred in only one (2.8%) case in group B (Table 2).

**Table 2:** Postoperative complications in both groups.

	**G I (N=33)**		**G II (N=35)**		**X^**2**^**	**P-value**
	**N**	**%**	**N**	**%**		
UCF	2	6	6	17.1	0.341	0.023^*^
Dehiscence of the glans	2	6.1	5	14.2	0.608	0.435
MS	1	3	8	22.9	5.814	0.016^*^
MR	2	6.1	1	2.9	0.413	0.520

* *Significant*.

*UCF, urethro-cutaneous fistula; MS, meatal stenosis; MR, meatal regression*.

The effect of the glans width as an index of the size of the glans on both urethral and glanular complications was statistically significant when correlated to both URF and meatal stenosis and non-significant if correlated with either glanular dehiscence or metal regression (Table 3).

**Table 3:** The effect of the glans size on the postoperative complications in both groups.

	**OR (95% CI)**	***P*-value**
UCF	3.500 (1.383–7.879)	0.007^*^
Dehiscence of the glans	2.001 (0.341–1.729)	0.209
MS	9.481 (1.114–12.669)	0.009^*^
MR	0.456 (0.039–5.279)	0.137

* *Significant*.

*UCF, Urethro-cutaneous fistula; MS, meatal stenosis; MR, meatal regression*.

The results of both the groups were evaluated using the HOSE 3 months postoperatively and is summarized in Table 4.

**Table 4:** Evaluation of the results using HOSE in both groups.

**HOSE**	**Group A (*n* = 33)**	**Group B (*n* = 35)**	***p*-value**
16	26 (78.8%)	28 (80%)	0.231
15	3 (9%)	4 (11.4%)	0.472
11	4 (12.2%)	3 (8.6%)	0.512

*HOSE, hypospadias objective score evaluation*.

## Discussion

A broad variety of techniques are used for the management of distal hypospadias, but none of them are the ideal surgical technique. The technique chosen depends more on the surgeon's experience and preference. During the last few years, TIPU has gained more acceptance among pediatric surgeons. However, many centers including our center, have mastered and consider Mathieu urethroplasty to be the technique of choice. One of the limitations associated with the use of the Mathieu urethroplasty is that the small size of the glans causes the housing of the neourethra to be done under severe tension leading to an increase in the rate of failure, but a deep incision of the urethral plate during the Mathieu urethroplasty markedly improves the capacity of the small glans to house the neourethra without tension (3, 6, 7).

Some studies tried to report different anatomical and surgical variables that may affect the prognosis of distal hypospadias management. These factors included the urethral plate characteristics, penile size, size of the glans, the use of a fine suture material, and the way in which the neourethra was fashioned (8).

We studied the prognosis of managing patients with distal hypospadias and a small glans using either HMU (group A) or TIPU (group B) with regards to the incidence of UCF, meatal stenosis, meatal regression and glanular dehiscence.

According to our results, the incidence of both UCF and meatal stenosis were significantly lower in group A than in group B. This may be attributed to the effect of increasing the diameter of the fashioned neourethra in group A, which resulted from the deep incision of the urethral plate, in contrast to the neourethra in group B. One of the major side effects of the classic Mathieu procedure was the meatal regression. The incidence of this complication during our study was 6% (2/33) in patients treated using HMU. This mainly was due to the maturation of the tip of the neourethra to the glans which prevented its regression.

Although the occurrence of glanular dehiscence was not significantly different between both the groups, we noticed a reduction of its rate in group A. The glanular wings in both the groups were adequately and extensively mobilized to eliminate the tension when closing the glans in the midline. By using logistic regression, we found that the risk of UCF increased by 12% for every 1.5 mm reduction in the width of the glans, while the risk of meatal stenosis increased by 25% for every 0.7 mm reduction in the width of the glans in group A and group B, respectively. This might reflect on the role of the incision of plate in HMU to improve the prognosis of repair in small sized glans.

Typically, the management of patients with a small glans included waiting until they reached puberty, following which testosterone was used (cream or systemic injection) or augmentation of the glans by modifying glansplasty was done. To overcome the small glans, Snodgrass et al. performed and advised extensive mobilization of the glanular wings to achieve a tension-free closure of the glans in the midline during TIPU. However, they found no significant effect on the complications developed in patients after the repair of distal hypospadias with the use of testosterone (9).

Bush et al. concluded that a glans size of <14 mm presented as a risk factor for both neourethra and glans complications after the repair of distal hypospadias. They found that even adequate mobilization of the wings of the glans remained insufficient to reduce these complications. They showed that the risk of complications increased by every 1 mm reduction in the width of the glans. Hence, preoperative assessment of the width of the glans reduced the complications to a great extent (10).

On the other hand, Faasse et al. reported that the small width of the glans had no effect on the prognosis of the repair of distal hypospadias. They found non-significant differences between groups with and without preoperative testosterone. They demonstrated through subgroup analysis that the occurrence of glanular complications other than occurrence of UCF may be related to the size of the glans.

Da Silva et al. studied the deferent anthropometric and biometric characteristics of the penis in patients with distal hypospadias. They found that although there were significant differences between the healthy and patients, these differences didn't have any effect on the prognosis of distal hypospadias repair. Therefore, the type of repair and efficiency of the surgical team may have the greatest impact on the prognosis (11).

To repair distal hypospadias with a narrow plate or a small glans using TIPU, Kolon and Gonzales grafted the incised plate with a dorsal lay graft from the inner aspect of the prepuce. They emphasized that it might widen the plate and allow closure of the glans without tension. The direct result of that modification was a reduction in the rate of meatal stenosis and UCF development. Since then, many reports have been studying about the grafting of the urethral plate and a debate is still present with regards to its feasibility, technical demands, prolonged operative time, and the incidence of complications. Some studies reported a positive effect of grafting on the results while others showed an improvement in the prognosis (12–14).

Theoretically, our HMU modification is considered a flapping of the incised plate, thus avoiding its narrowing during tubularization, increasing the diameter of the neourethra, and adding a considerable width to the glans. This acts on adequate mobilization of the glanular wings to avoid tension closure of the glans and decrease any glanular dehiscence in group A.

The fact that our study was not a multi-center study and that the duration of the follow-up was only up to 8 months was a limitation. Additionally, there is still a strong need to build up prognostic modules of glans characters to predict the success of complications following distal hypospadias repair.

## Conclusion

Distal hypospadias with a small glans (<14 mm in width) presented a challenge during repair. We recommend the use of HMU as it was associated with a lower incidence of UCF, meatal stenosis, and dehiscence of the glans when compared to TIPU.

## Data Availability Statement

The original contributions presented in the study are included in the article/supplementary material, further inquiries can be directed to the corresponding author/s.

## Ethics Statement

The studies involving human participants were reviewed and approved by Institutional Review Board with the approval code 34403/1/21. The trial was registered in clinicaltrials.gov registry under identifier NCT04946058 clinicaltrials.gov/ct2/show/NCT04946058. Written informed consent to participate in this study was provided by the participants' legal guardian/next of kin.

## Author Contributions

All authors listed have made a substantial, direct, and intellectual contribution to the work and approved it for publication.

## Conflict of Interest

The authors declare that the research was conducted in the absence of any commercial or financial relationships that could be construed as a potential conflict of interest.

## Publisher's Note

All claims expressed in this article are solely those of the authors and do not necessarily represent those of their affiliated organizations, or those of the publisher, the editors and the reviewers. Any product that may be evaluated in this article, or claim that may be made by its manufacturer, is not guaranteed or endorsed by the publisher.

## References

[1] Karabulut A Sunay M Erdem K Emir L Erol D. Retrospective analysis of the results obtained by using Mathieu and TIP urethroplasty techniques in recurrent hypospadias repair. J Pediatr Urol. (2008) 4:359–63. 10.1016/j.jpurol.2008.02.007 18790420

[2] Zhang Y Shen Z Zhou X Chi Z Hong X Huang Y. Comparison of meatal-based flap (Mathieu) and tubularized incised-plate (TIP) urethroplasties for primary distal hypospadias: a systematic review and meta-analysis. J Pediatr Surg. (2020) 55:2718–27. 10.1016/j.jpedsurg.2020.03.013 32439182

[3] Khirallah M Eldessouki I. Hybrid Mathieu urethroplasty: a simple modification outcomes. Res Rep Urol. (2021) 13:473–8. 10.2147/RRU.S318900 34262885 PMC8275146

[4] Bush NC Dajusta D Snodgrass W. Glans penis width in patients with hypospadias compared to healthy controls. J Pediatr Urol. (2013) 9:1188–91. 10.1016/j.jpurol.2013.05.004 23768835

[5] Holland AJA Smith GH Ross FI Cass DT. HOSE: an objective scoring system for evaluating the results of hypospadias surgery. BJU Int. (2000) 88:255–8. 10.1046/j.1464-410x.2001.02280.x 11488741

[6] Wilkinson DJ Farrelly P Kenny SE. Outcomes in distal hypospadias: a systemic review of the mathieu and tubularized incised plate repairs. J Pediatr Urol. (2012) 8:307–12. 10.1016/j.jpurol.2010.11.008 21159560

[7] Furan W Yinghua X Hongji Z. Systematic review and meta-analysis of students comparing the perimeatal-based flap and tubularized incised-plate techniques for primary hypospadias repair. Pediatr Surg Int. (2013) 29:811–21. 10.1007/s00383-013-3335-3 23793987

[8] Eassa W Jednak R Capolicchio JP Brzezinski A El-Sherbiny M. Risk factors for reoperation following tubularized incised plate urethroplasty: a comprehensive analysis. Pediatr Urol. (2011) 3:716–20. 10.1016/j.urology.2010.07.467 20970827

[9] Snodgrass WT Villanueva C Granberg C Bush NC. Objective use of testosterone reveals androgen insensitivity in patients with proximal hypospadias. J Pediatr Urol. (2014) 10:118–22. 10.1016/j.jpurol.2013.07.006 23962431

[10] Bush NC Villanueva C Snodgrass W. Glans size is an independent risk factor for urethroplasty complications after hypospadias repair. J Pediatr Urol. (2015) 11:355.e1–e5. 10.1016/j.jpurol.2015.05.029 26320396

[11] Da Silva EA Lobountchenko T Marun MN Damiao R. Role of penile biometric characteristics on surgical outcome of hypospadias repair. Pediatr Surg Int. (2014) 30:339–44. 10.1007/s00383-013-3442-1 24374664

[12] Silay MS Hoten L Bhatt N Quaedackers J Bogaert G Dogan HS et al. Are there any benefits of using an inlay graft in the treatment of primary hypospadias in children? A systematic review and metanalysis. J Pediatr Urol. (2021) 17:303–15. 10.1016/j.jpurol.2021.02.013 33691984

[13] Eldeeb M Nagla S Abou-Farha M Hassan A. Snodgrass vs snodgraft operation to repair the distal hypospadias in the narrow urethral plate. J Pediatr Urol. (2020) 16:165.e1–e8. 10.1016/j.jpurol.2020.01.006 32144015

[14] Gupta V Yadav SK Alanzi T Amer I Salah M Ahmed M. Grafted tubularised incised plate urethroplasty: an objective assessment of outcome with lessons learnt from surgical experience with 263 cases. Arab J Urol. (2016) 14:299e304. 10.1016/j.aju.2016.09.002 27900221 PMC5122801

